# The Latest Developments of National Insurance

**Published:** 1914-01-17

**Authors:** 


### The Latest Developments of National Insurance.

### Drugs and Prescriptions.

The working of the National Insurance Act has already falsified many of the actuarial and other estimates upon which the provisions of the Act were framed. In no section of its operations has this been more noticeable than in regard to drugs and prescriptions, and a whole crop of instances in point have come to light within the last few days.

The original financial allocation for meeting the cost of drugs for the insured was one and sixpence per patient per annum, with an extra sixpence in "suspense," which was to go to the doctors if the drug fund (at eighteenpence) proved sufficient, but to the chemists if insufficient. The object of this provision was quite laudable in itself, for it was simply the encouragement of economical prescribing. It failed, however, to take into consideration the fact that doctors are human beings, and that therefore the only way to secure economy in drugs is to give each individual panel doctor a direct interest in such economies as he can effect in his own practice, not in those of other doctors; and it was further vitiated by the serious under-estimation of the amount of sickness which was likely to prevail amongst the insured. In short, the whole two shillings has gone to the chemists, who even then are in many districts not receiving a living wage out of Insurance dispensing.

To compel panel doctors to study economy, different Insurance Committees are devising various measures, some of which are salutary, others very much the reverse. At Sheffield, for example, after some preliminary wrangling, the doctors and chemists have met in conference, and the former have agreed not to prescribe secret remedies and proprietary articles. This is excellent, and they have also agreed to be as economical in their prescriptions as the needs of insured will allow; it only remains to be hoped that this sensible method of surmounting the difficulty of the drug fund will work better than the stringent and derogatory rules which some other Insurance Committees are attempting to impose.

### A Stupid Restriction.

At Manchester the panel doctors are receiving instructions which are, in the main, similar to the arrangements at Sheffield. But there is one clause which is unjust alike to patient and practitioner. It enacts that extract of malt and cod-liver oil shall not be prescribed except in tuberculous cases notified as such. This extraordinarily stupid order cuts off just the very class of suspects and "pre-tuberculous" persons for whom these drugs are of proved value. The indubitable consumptive can (if he is lucky) get sanatorium benefit of a kind, whereas those in whom the disease has not yet got a hold, or in whom its presence cannot be definitely diagnosed, are dependent more upon malt and oil for the fortification of their resisting powers. Another Manchester innovation is the decision to limit to £800 the income of each individual paneller. If this means that no doctor is allowed to be responsible for more than 2,300 of the insured, it may be said that a limit of some sort is a crying need, but that it would be preferable to fix it lower still.

In the Home Counties great indignation has been aroused by the economies which one Insurance Committee is effecting at the expense of the insured. A good many consumptives have been receiving "domiciliary" treatment instead of the accommodation in the first-class hotels which Mr. George used to talk about. Part of this treatment has been a supply of "ancillary nourishment" in the shape of milk and eggs. This is now to be discontinued from motives of economy, and great dissatisfaction has not unnaturally been voiced in the public press on behalf of the insured.

### The Edinburgh Royal Infirmary.

The annual report of this, the premier voluntary hospital in Scotland, was presented by the Board of Managers to the annual meeting of subscribers on January 5 last. It illustrates very well the dependence of the National Insurance Act and of the health of the insured classes upon the voluntary hospital system; and the heavy cost which is thrown upon the hospitals by the admission of insured persons. During the eleven months ending December 31, 1913, the patients admitted to the wards numbered 11,691. Of these nearly 44 per cent. were insured persons, and the cost of their treatment was well in excess of £20,000. During the same period more than 32 per cent. of the out-patients were insured persons—a proportion so high that we can only conclude that the "canny" Scot prefers the out-patient clinic to his panel doctor, and that insufficient pains are taken to exclude those cases which the panel doctor ought to be able to treat. The Approved Societies and the local Insurance Committee have been invited to ponder over the heavy cost to the infirmary of treating their cases, and to subscribe towards the funds of the institution; but their reply has not yet been received. Meanwhile the only recoupment which the Edinburgh Infirmary has had towards this heavy burden consists in freewill offerings from insured persons totalling £21.

